# Novel protein chip for the detection of antibodies against infectious bronchitis virus

**DOI:** 10.1186/s12917-018-1586-x

**Published:** 2018-09-17

**Authors:** Liping Yan, Jianhua Hu, Jing Lei, Zhiyu Shi, Qian Xiao, Zhenwei Bi, Lu Yao, Yuan Li, Yuqing Chen, An Fang, Hui Li, Suquan Song, Min Liao, Jiyong Zhou

**Affiliations:** 1 MOE Joint International Research Laboratory of Animal Health and Food Safety, Institute of Immunology and College of Veterinary Medicine, Nanjing Agricultural University, Nanjing, 210095 People’s Republic of China; 2 Jiangsu Engineering Laboratory of Animal Immunology, Institute of Immunology and College of Veterinary Medicine, Nanjing Agricultural University, Nanjing, 210095 People’s Republic of China; 3 Jiangsu Detection Center of Terrestrial Wildlife Disease, Institute of Immunology and College of Veterinary Medicine, Nanjing Agricultural University, Nanjing, 210095 People’s Republic of China; 4 Key Laboratory of Animal Virology, Ministry of Agriculture, Zhejiang University, Hangzhou, 310058 People’s Republic of China; 5 Collaborative Innovation Center for Diagnosis and Treatment of Infectious Diseases, The First Affiliated Hospital, Zhejiang University, Hangzhou, 310058 People’s Republic of China

**Keywords:** Infectious bronchitis, Protein chip, Antibody detection

## Abstract

**Background:**

Infectious bronchitis (IB) caused by the IB virus (IBV) can cause acute damage to chickens around the world. Therefore, rapid diagnosis and immune status determination are critical for controlling IBV outbreaks. Enzyme-linked immunosorbent assays (ELISAs) have been widely used in the detection of IBV antibodies in the early infection and continuous infection of IB because they are more sensitive and quicker than other diagnostic methods.

**Results:**

We have developed two indirect microarray methods to detect antibodies against IBV: a chemiluminescent immunoassay test (CIT) and a rapid diagnostic test (RDT). IBV nonstructural protein 5 (nsp5) was expressed, purified from *Escherichia coli*, and used to spot the initiator integrated poly(dimethylsiloxane), which can provide a near “zero” background for serological assays. Compared with the IDEXX IBV Ab Test kit, CIT and RDT have a sensitivity and specificity of at least 98.88% and 91.67%, respectively. No cross-reaction was detected with antibodies against avian influenza virus subtypes (H5, H7, and H9), Newcastle disease virus, Marek’s disease virus, infectious bursal disease virus, and chicken anemia virus. The coefficients of variation of the reproducibility of the intra- and inter-assays for CIT ranged from 0.8 to 18.63%. The reproducibility of RDT was consistent with the original results. The application of the IBV nsp5 protein microarray showed that the positive rate of the CIT was 96.77%, that of the nsp5 ELISA was 91.40%, and that of the RDT was 90.32%. Furthermore, the RDT, which was visible to the naked eye, could be completed within 15 min. Our results indicated that compared with nsp5 ELISA, the CIT was more sensitive, and the RDT had similar positive rates but was faster. Furthermore, the two proposed methods were specific and stable.

**Conclusions:**

Two microarray assays, which were rapid, specific, sensitive, and relatively simple, were developed for the detection of an antibody against IBV. These methods can be of great value for the surveillance of pathogens and monitoring the efficiency of vaccination.

## Background

Infectious bronchitis (IB) is an acute, highly contagious, and economically important respiratory disease in chickens; it is caused by the IB virus (IBV), which is a significant respiratory pathogen that causes considerable economic losses in the commercial poultry industry worldwide [1]. The IBV genome is a single-stranded, positive-sense RNA that is 27.6 kb in size [2]. It encodes four major structural proteins, namely, glycosylated spike protein (S), membrane protein (M), phosphorylated nucleoprotein (N), and envelope protein (E) [3], and 15 nonstructural proteins (nsp2–nsp16). Generally, nonstructural proteins are present in infected cells but not in the virus, and they only play a role in the process of virus infection and replication [4]. Chickens immunized with an inactivated vaccine will produce no antibodies or low levels of antibodies against viral nonstructural proteins. Thus, nonstructural proteins have the potential application in differentiating natural infection from inactivated vaccine immunity [5].

IB diagnosis is complicated due to the continual emergence of new serotypes [6] and the difficulty in differentiating IB from other upper respiratory diseases [7]. Virus isolation is regarded as the gold standard for the diagnosis of IBV infection, but it is time-consuming and costly [8]. The agar gel precipitation test is used in IBV antibody detection; however, this method has low sensitivity. Hemagglutination inhibition (HI) assays are suitable for the rapid diagnosis of IB, which requires a series of methods to treat the antigen; however, the HI titer is not related to protection. The virus neutralization test correlates with protection and has the highest specificity among IB diagnostic methods, but it is tedious and laborious [9].

Compared with these methods, enzyme-linked immunosorbent assay (ELISA) has been widely used for testing IBV early infection and continuous infection, and this technique can be used for both antigenic and antibody detection. The immunogenicity of the coating antigen is one of the crucial factors when performing an ELISA test for antibody detection. An inactivated whole virus is the most commonly used coating antigen in commercial diagnosis kits for IBV diagnosis. Recombinant antigenic protein expressed using prokaryotic, yeast, or baculovirus systems has been widely used in preparing specific coating antigens for ELISA kits [10–13]. ELISAs based on purified recombinant protein may have higher specificity and sensitivity as the target antigen is immune-dominant and devoid of any nonspecific immune responses [14]. ELISAs based on whole virus particles as well as recombinant S1 (spike protein 1 subunit) and N proteins (nucleoproteins) can provide a rapid and large-scale detection method for IBV infection. However, few IBV detection methods have been developed based on nonstructural proteins (nsps). Our laboratory has established an nsp5 ELISA to detect IBV infection [4]. The nsp5 antibodies detected are likely to be non-neutralizing and exist in lower numbers than the ones generated by other proteins. Based on previous studies, we developed a rapid, highly sensitive protein microarray and a visible detection method to detect IBV nsp5 antibodies for epidemiological investigation and antibody level monitoring.

## Methods

### Reagents

Initiator integrated poly(dimethylsiloxane) (iPDMS) membrane 26 (15 × 15 mm^2^) was obtained from BS Company (Zhejiang, China). 1-Ethyl-3-(3-dimethylaminopropyl) carbodiimide (EDC) and N-hydroxysuccinimide (NHS) were purchased from Medpep (Shanghai, China). Chicken IgY was purchased from SouthernBiotech (Birmingham, UK). Horseradish peroxidase-labeled goat anti-chicken IgY (HRP-IgG) was obtained from KPL (Dianova, USA). Peroxidase conjugate stabilizer/diluent and chemiluminescent substrate (SuperSignal ELISA Femto Maximum Sensitivity Substrate) were purchased from Thermo Fischer (Massachusetts, USA). Tetramethylbenzidine (TMB) chromogenic reagent was purchased from Nanjing Jiancheng Bioengineering Institute, China. Marek’s disease virus (MDV) was purchased from Harbin National Engineering Research Center of Veterinary Biologics Corp (Harbin, China).

### Serum samples

In this study, 328 clinical serum samples were collected from a chicken farm. Forty-two negative sera were obtained from different ages of specific-pathogen-free (SPF) chickens raised in SPF isolators in Zhejiang University.

Three-month-old SPF chickens, which were purchased from Shennong Company (Zhejiang, China) and reared in SPF isolators, were used to prepare negative serum and positive serum. We prepared standard positive serum samples from chickens infected with H5, H7, and H9 avian influenza virus (AIV); Newcastle disease virus (NDV); IBV; infectious bursal disease virus (IBDV); and chicken anemia virus (CAV).

### Protein chip microarray preparation

A microarray was prepared in a 100,000-grade clean room. Proteins were first dissolved with 30% acetonitrile solution (*v*/v, in Milli-Q water) to 1 mg/mL stock solution and then diluted into the optimized concentration (200 μg/mL) with printing buffer (0.3 M phosphate buffer, 0.2% glycerin, 0.01% Triton X-100, and 1.5% mannitol) for further printing. iPDMS membranes were first activated with 0.1 M EDC and 0.1 M NHS mixtures for 30 min, rinsed with Milli-Q water, and immediately used for printing. To determine the optimal antigen concentration, the protein was diluted with 0.3 M phosphate buffer to different concentrations. Each dilution of protein was printed on iPDMS using a protein microarray (SCIENION, Germany). Once the antigen concentration was determined, the optimized concentration of nsp5 was achieved by dilution with printing buffer and printed on iPDMS for subsequent experiments in triplicate. The protein microarray was prepared using the SmartArrayer 48 contact printer (Capitalbio, China) with approximately 0.6 nL of printing solution for each sample. Each subarray had a positive control with chicken-IgY at a concentration of 0.1 mg/mL and negative control with printing buffer.

### Establishment of the chemiluminescent immunoassay test (CIT)

**Fig. 1:**
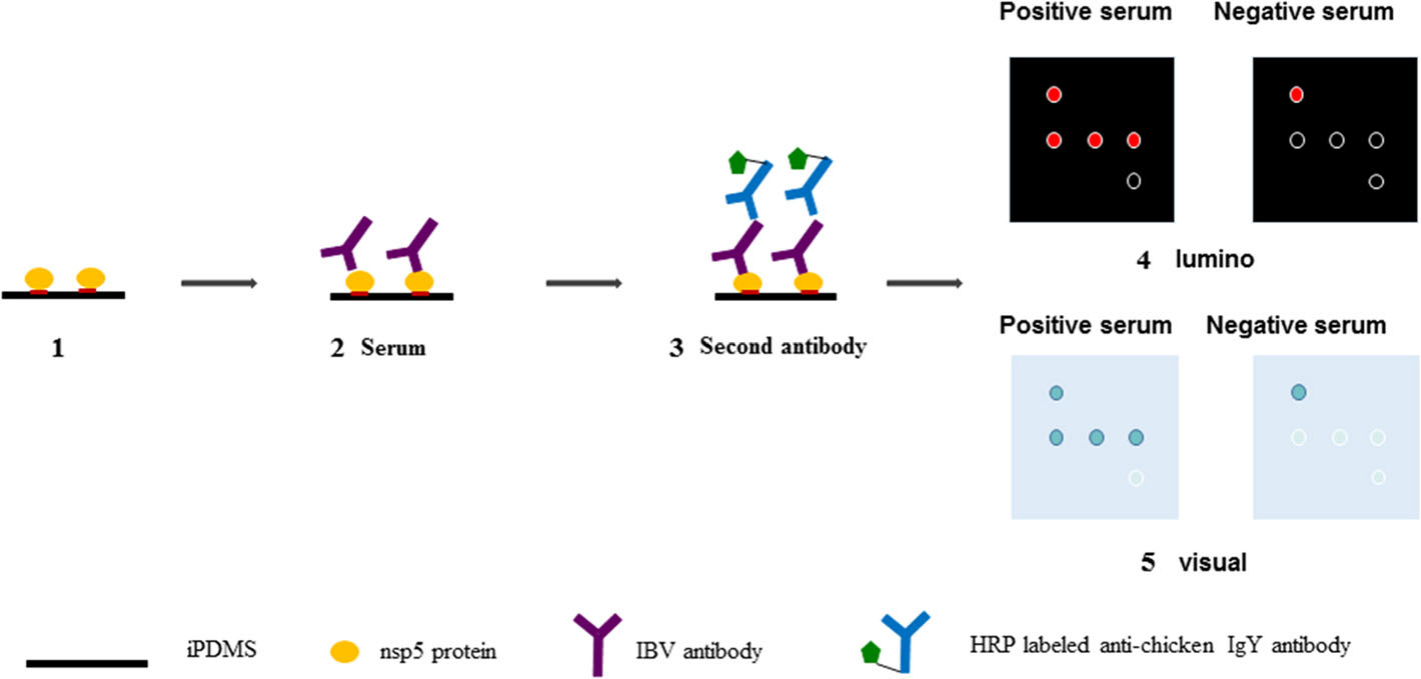
Schematic illustration of the protein microarrays for the detection of antibodies against IBV. Step 1, the prepared chip was rinsed thrice with PBST; Step 2, 100 μL of diluted serum was added and incubated on a constant temperature oscillator and then washed with PBST thrice; Step 3, 100 μL of goat anti-chicken IgY conjugated to HRP was added, and the plate was incubated on a constant temperature oscillator and washed with PBST thrice; Step 4, for chemiluminescence, 15 μL of chemiluminescent substrate was added to each well, and images were taken at a wavelength of 645 nm with Amersham Imager 600; Step 5, for RDT, 60 μL of TMB was added to each well and incubated for 5 min in a dark place; then, the results were observed

The procedure for the CIT is shown in Fig. 1. Serum samples were first diluted with serum-dilution buffer (1% bovine serum albumin, 1% casein, 0.5% sucrose, 0.2% polyvinylpyrrolidone, 0.5% Tween 20 in 0.01 M phosphate-buffered saline, pH = 7.4). In total, 100 μL of the diluted serum samples was then added into each protein microarray and incubated for 30 min on a shaker (Thermo Fischer, USA) at 500 rpm and 37 °C. Microarrays incubated with serum-dilution buffer were used as negative controls. Each microarray was then rinsed thrice with washing buffer and incubated with 100 μL of 1 mg/mL HRP-IgG diluted 1:20,000 in peroxidase conjugate stabilizer/diluent for another 30 min on the shaker (500 rpm, 37 °C), followed by the same washing steps described above. A total of 15 μL of the chemiluminescent substrate was added to the microarray, and images were taken at a wavelength of 645 nm with the Amersham Imager 600 (GE, USA). Chemiluminescent signals were acquired using GenePix Pro 6.0 software, and the signal-to-noise ratio (SNR) was calculated.

The purified recombinant nsp5 was printed on the iPDMS membrane to form a microarray with a concentration of 0.05, 0.1, 0.2, and 0.4 mg/mL. Subsequently, the serum samples were added to microplates at the following dilutions: 1:100, 1:200, 1:400, 1:600, 1:800, 1:1600, 1:3200, and 1:6400. To identify the optimal time of exposure, the images were taken at an exposure time of 30 s, 1 min, 2 min, 3 min, and 4 min.

To determine the CIT threshold, a total of 184 serum samples, including 142 positive samples and 42 negative samples, identified by the IDEXX IBV Ab Test kit were tested according to the optimal working conditions. Results were then compared with those obtained using the IDEXX IBV Ab Test kit. Finally, receiver operating characteristic (ROC) curve analysis was conducted to determine the accuracy of the IBV protein microarray test.

The specificity of the CIT was evaluated by detecting the positive sera against AIV (H5, H7, and H9), NDV, MDV, IBDV, and CAV.

The evaluation of the CIT reproducibility within and between runs was carried out as described by Jacobson [15]. Thirteen field serum samples (nine IDEXX positive samples and four IDEXX negative samples) were selected for the reproducibility experiments. For intra-assay reproducibility, three replicates of each serum sample were analyzed within the same plate. For inter-assay reproducibility, three replicates of each sample were run in different plates. The mean SNR, standard deviation (SD), and coefficient of variation (CV) were then calculated.

### Development of the rapid diagnostic test (RDT)

The procedure of the RDT is also shown in Fig. 1. Serum was first diluted 1:100 with serum-dilution buffer, and 100 μL of the diluted serum sample was added into each protein microarray and incubated for 5 min on a shaker (500 rpm, 37 °C). The microarray incubated with serum-dilution buffer was used as a negative control. The microarray was then rinsed thrice with washing buffer and incubated with 100 μL of 1 mg/mL HRP-IgG diluted 1:2000 in peroxidase conjugate stabilizer/diluent for another 5 min on a shaker (500 rpm, 37 °C), followed by the same washing steps described above. A total of 60 μL of TMB was added to the microarray and incubated for 5 min in the dark; then, the results were observed.

To confirm the concentration of the nsp5 protein in the RDT, the purified recombinant nsp5 was printed on an iPDMS membrane to form a microarray with concentrations of 0.05, 0.1, 0.2, and 0.4 mg/mL. The specificity of the RDT was evaluated by detecting the positive sera against AIV (H5, H7, and H9), NDV, MDV, IBDV, and CAV. The sensitivity experiments of the RDT were conducted by detecting the IBV positive serum with different titers. Then, the results were observed, and the detection limit was determined.

### Application of the CIT and the RDT

To further evaluate the CIT and RDT, 186 clinical serum samples were detected by the CIT, RDT, and nsp5 ELISA antibody test kit [4]. Subsequently, the positive rate of each method was determined.

### Statistical analysis

Chemiluminescent signals were acquired using GenePix, and the SNR was calculated as follows: SNR = (Signal intensity − Background)/Background. GraphPad Prism 6 and Microsoft Excel were used for the statistical analysis of all data, including the determination of the threshold and the calculation of the SNR value, means, SDs, and CVs. The ROC curve was obtained using GraphPad Prism 6. Sensitivity and specificity were calculated according to the following formulas: Sensitivity = True positive/(True positive + False negative) × 100%; Specificity = True negatives/(False positives + True negatives) × 100%. The area under the curve (AUC) was used to validate the diagnostic application of the CIT. The area under the ROC curve quantifies the overall ability of the test to discriminate between those individuals with the disease and those without the disease. A truly useless test (one no better at identifying true positives than flipping a coin) has an AUC of 0.5, whereas a perfect test (one that has zero false positives and zero false negatives) has an AUC of 1.

## Results

### Establishment of the CIT

**Fig. 2:**
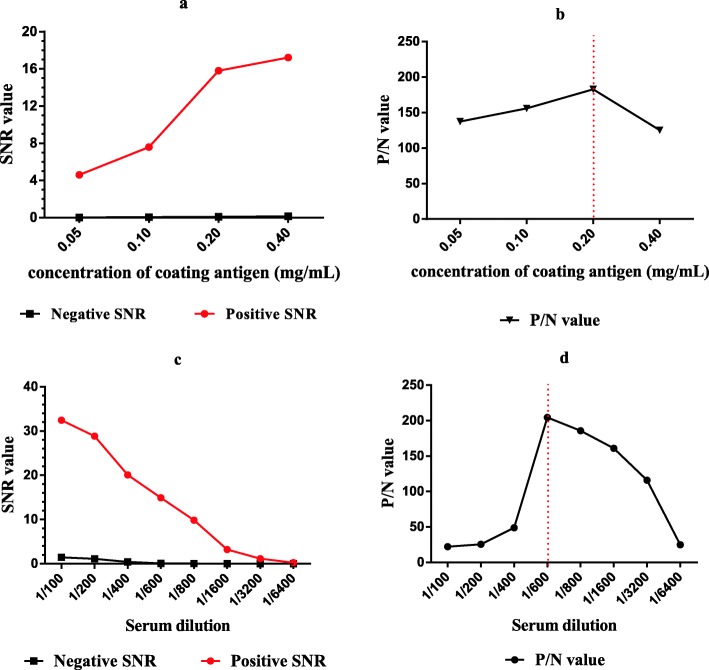
Optimization of the microarray working conditions. **a** SNR variation across different IBV nsp5 concentrations (0.05, 0.1, 0.2, and 0.4 mg/mL) of the coating antigen. **b** P/N value between the positive and negative SNRs with different IBV nsp5 concentrations. **c** SNR variation across different serum dilutions. **d** P/N value between the positive and the negative SNRs with the optimal dilution of the serum sample (1:600)

**Fig. 3:**
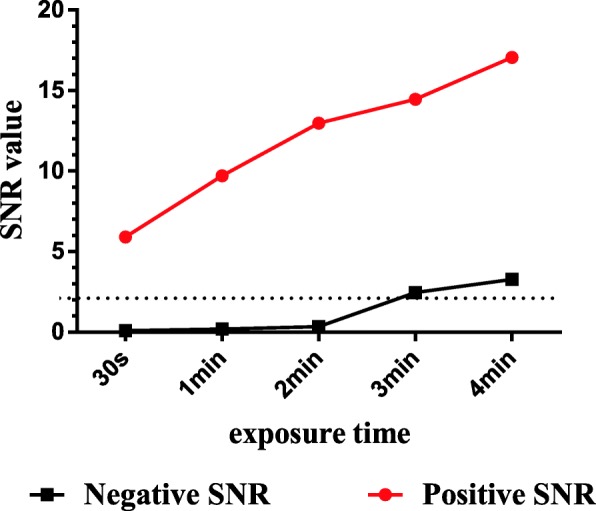
SNR changes with different exposure times

For the CIT, the optimal antigen concentration was 0.2 mg/mL (Fig. 2a, b), and the dilution for the serum samples was 1:600 (Fig. 2c, d), on the assumption that the SNR between the positive and the negative sera was the highest. The dilution of the HRP-conjugated goat anti-chicken antibody was defined as 1:20,000. When the exposure time was more than 2 min, the SNR of the negative serum rose rapidly; thus, we set the exposure time to 2 min (Fig. 3).

**Fig. 4:**
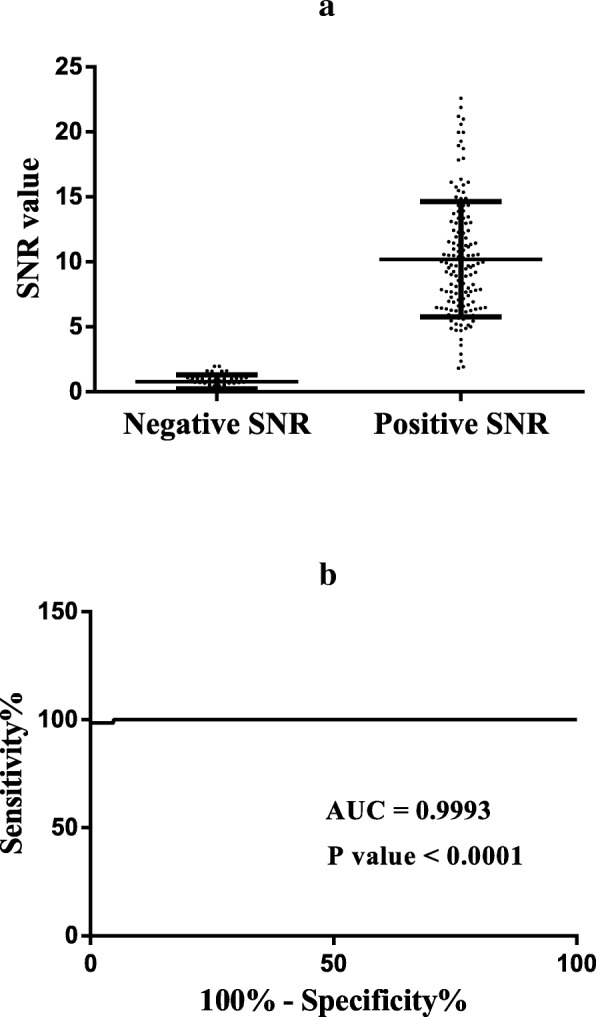
**a** Distribution of the SNRs of the IDEXX-positive (*n* = 142) and IDEXX-negative (*n* = 42) serum samples of the clinical sera obtained from the IBV protein microarray. The threshold was defined as 2. The diagnostic sensitivity and specificity of the assay were greater than 90%. **b** ROC curve obtained with GraphPad Prism 6 software with positive (*n* = 142) and negative (*n* = 42) samples. The AUC was 0.9993, indicating that the IBV protein microarray is a reliable test

ROC analysis showed that the IBV nsp5 microarray had high selectivity (*p* < 0.0001) between the positive and the negative samples, and the AUC was 0.9993 (Fig. 4a). Based on the ROC analysis of the IBV nsp5 microarray, the SNR value of the IDEXX-negative serum samples varied from a minimum of 0.01 to a maximum of 1.964, whereas the SNR value of the IDEXX-positive serum samples was from a minimum of 1.82 to a maximum of 23.59 (Fig. 4b). A threshold SNR value of 2 for IBV nsp5 microarray was found to provide optimal results, with a sensitivity of 98.59%, a specificity of 100%, and an accuracy of 98.91% compared with the results of other thresholds (Table 1). Thus, the samples with SNR < 2 were considered negative, whereas those with SNR ≥ 2 were considered positive.

**Table 1:** Evaluation of the IBV protein microarray with selected thresholds

Threshold	Sensitivity (%)	Specificity (%)	Accuracy (%)
> 1.940	98.59	95.24	98.91
> 1.962	98.59	97.62	98.91
> 2	98.59	100	98.91
> 2.635	97.89	100	98.3

The specificity of the CIT was evaluated by detecting the cross-reactivity of the antibodies against AIV (H5, H7, and H9), NDV, MDV, IBDV, and CAV. The SNRs of all sera from the previously mentioned viruses were all below the threshold of 2. These data revealed that no cross-reactivity occurred between the IBV GST-fused nsp5 antigen and antibodies against other avian viruses. This result demonstrated that the antigen has a high specificity.

The reproducibility of the CIT detection was determined by comparing the SNR value of each clinical serum sample from the below tests. The within-plate CVs of nine positive and four negative serum samples tested ranged from 0.8 to 18.63% (Table 2), whereas the between-run CVs of these serum samples ranged from 1.89 to 18.01% (Table 3). These results showed that the CIT detection results were reproducible and had low and acceptable variation.

**Table 2:** CVs of positive sera within the same run

No.	I (SNR value)	II (SNR value)	III (SNR value)	X (Mean)	SD	CV (%)
1	0.69	1.02	0.72	0.81	0.15	18.63
2	1.64	1.80	1.30	1.58	0.21	13.22
3	0.26	0.30	0.29	0.28	0.02	6.80
4	0.01	0.00	0.00	0.00	0.00	17.78
5	9.40	9.94	7.43	8.92	1.08	12.09
6	14.58	17.49	18.49	16.85	1.66	9.84
7	15.43	15.42	15.69	15.51	0.12	0.80
8	14.57	14.46	15.09	14.70	0.28	1.87
9	21.88	19.47	19.34	20.23	1.17	5.78
10	14.16	13.74	14.00	13.96	0.17	1.24
11	21.52	23.23	22.00	22.25	0.72	3.24
12	17.42	15.34	15.70	16.15	0.91	5.61
13	19.11	20.15	20.55	19.94	0.61	3.06

**Table 3:** CVs of positive and negative sera between runs

No.	I (SNR value)	II (SNR value)	III (SNR value)	X (Mean)	SD	CV (%)
1	0.70	0.69	0.72	0.70	0.01	1.89
2	2.00	1.80	1.70	1.83	0.12	6.73
3	0.30	0.30	0.34	0.31	0.02	6.06
4	0.01	0.01	0.01	0.01	0.00	14.48
5	3.08	2.07	2.24	2.46	0.44	18.01
6	15.94	17.49	16.86	16.76	0.64	3.80
7	13.16	11.57	14.70	13.14	1.28	9.73
8	14.57	15.09	13.69	14.45	0.58	4.00
9	21.88	22.33	19.47	21.22	1.26	5.92
10	11.88	13.61	14.38	13.29	1.04	7.86
11	17.56	15.89	14.24	15.90	1.35	8.52
12	11.83	13.78	14.42	13.34	1.10	8.25
13	13.96	17.50	14.80	15.42	1.51	9.80

### Development of the RDT

One hundred and forty-four clinical serum samples (130 samples were positive for antibodies against IBV, and 14 samples were negative as confirmed by the IDEXX IBV Ab Test kit) were subjected to visual rapid detection following the procedure described above. The data showed that 130 serum samples were positive for antibodies against IBV, and 14 samples were negative, similar to the results of the IDEXX IBV Ab Test kit with the nsp5 concentration of 0.2 mg/mL (Table 4). If IBV antibodies exist in the serum, the spot with the IBV antigen turns blue, thereby allowing us to determine the concentration of nsp5 as 0.2 mg/mL.

**Table 4:** Comparison of the detection results at different antigen concentrations for the RDT

Antigen concentration	0.4 mg/mL	0.2 mg/mL	0.1 mg/mL	0.05 mg/mL
Positive number	134	130	129	127
Negative number	10	14	15	17

**Fig. 5:**
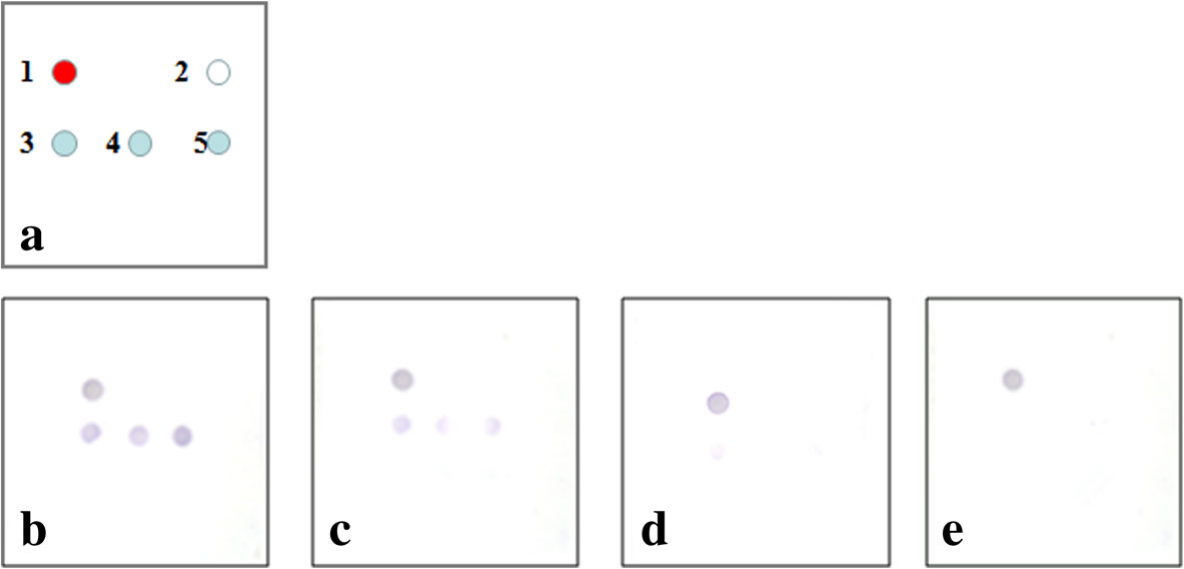
Sensitivity experiments of the RDT. **a** Array of the protein chip: 1, positive control (chicken IgY); 2, negative control; 3, 4, and 5, IBV nsp5 spots. **b** IBV positive serum diluted 1:100. **c** IBV positive serum diluted 1:1000. **d** IBV positive serum diluted 1:10,000. **e** Negative SPF chicken serum

The specificity of the RDT was evaluated by detecting the cross-reactivity of antibodies against AIV (H5, H7, and H9), NDV, MDV, IBDV, and CAV. The specific experiments of the RDT showed that no cross-reaction occurred between the IBV GST-fused nsp5 antigen and the antibodies against other avian viruses. The sensitivity experiments demonstrated that when the positive serum was diluted 1:1000, the spot still turned blue (Fig. 5).

### Application of the IBV nsp5 protein microarray

To further evaluate the IBV nsp5 microarray, 186 clinical serum samples were detected by the IBV nsp5 microarray and the nsp5 ELISA antibody test kit. The results showed that of the 186 samples, 170 samples were positive for antibodies against IBV, and 16 samples were negative according to the nsp5 ELISA kit. A total of 180 out of the 186 samples were positive, and 6 samples were negative according to the CIT. A total of 167 positive samples and 19 negative samples were detected by the RDT. The positive rate of the CIT was 96.77%, that of the nsp5 ELISA was 91.40%, and that of the RDT was 90.32% (Table 5).

**Table 5:** Comparison of IBV protein microarray and the nsp5 ELISA kit with 186 clinical serum samples

Samples	nsp5 ELISA	CIT	RDT
Positive	170	180	168
Negative	16	6	18
Positive rates	91.40%	96.77%	90.32%

## Discussion

Most serological assays, including the IDEXX ELISA kit, use viral particles of IBV as an antigen for the detection of antibodies against IBV. However, the preparation of purified virions for use as an antigen is time-consuming and expensive. In the present study, recombinant nonstructural proteins expressed in *Escherichia coli* antigen-based protein microarray was evaluated for the first time in the serological diagnosis of IB [4]. Protein microarrays have high sensitivity and good reproducibility in quantitative and qualitative assays, and they are a valuable asset when analyzing complex biological samples [16]. In clinical sample testing, many factors, including time, cost, accuracy, sensitivity, and throughput, determine the performance and usefulness of an immunoassay. In this study, a new solidly supported material, iPDMS membrane, which has a near “zero” background for identification, was used. It achieved high sensitivity in detecting antibodies in serum [17]. These unique features of iPDMS not only simplify data analysis but also reduce nonspecific interactions [18]. ELISA detection has been widely used in the detection of IBV antibodies in early infection and continuous infection of IB and vaccine-immune, and no diagnosis method is more sensitive and quicker than ELISA. In this study, two microarray methods (CIT and RDT) were established. Except for the method of the observation, the reaction processes of the two methods are akin to the detection process of ELISA. However, unlike ELISA, the established methods only require 2 ng of antigen coating on each spot, and the amount of HRP-IgG required for each reaction well is only 5 ng. The antigen and HRP-IgG used in both methods were less than those used in ELISA, thereby reducing the cost of detection. In addition, the CIT can detect antibodies against IBV nsp5 quantitatively and is more sensitive than the IBV nsp5 ELISA kit. The RDT was developed to detect antibodies against IBV visually, and the results can be obtained within 15 min with great sensitivity and specificity. Compared with ELISA, RDT has a shorter detection time and better detection efficiency. In this study, we only used one antigen of IBV for testing and verification. In the future, we will apply antigens of different diseases to iPDMS to achieve high-throughput test results.

For the establishment of the IBV nsp5 protein chip, we first optimized the procedure and determined the CIT threshold as 2 with the IDEXX IBV antibody detection kit. With the threshold of 2, the CIT showed high sensitivity (98.59%), specificity (100%), and accuracy (98.91%) in the antibody detection of the samples compared with those of other thresholds (Table 1). The RDT demonstrated a high success rate compared with the commercial IDEXX IBV Ab Test kit, suggesting that the RDT is a reliable assay for the detection of IBV infection. Clinical serum samples were also subjected to rapid detection. Furthermore, the RDT has higher sensitivity than the commercial IDEXX IBV Ab Test kit. It is also simpler and faster than ELISA methods. To further evaluate IBV nsp5 protein chip, 186 clinical serum samples were detected by the IBV nsp5 protein chip and the nsp5 ELISA antibody test kit. The positive rates of the CIT, nsp5 ELISA, and RDT were 96.77%, 91.40%, and 90.32%, respectively. Compared with nsp5 ELISA, the CIT was more sensitive, and the RDT had similar positive rates but was faster.

Protein chips are a high-throughput monitoring system that monitors the interaction among protein molecules through the interaction between a target molecule and a capture molecule. Although protein chips have been produced in the context of proteomics research, its application is not limited to proteomics alone. With the development of protein chip technology, researchers have gradually applied this technology to other fields, such as food inspection, disease diagnosis, drug screening, agriculture, forestry, animal husbandry, and forensic science. At present, this technology is rarely studied and applied in veterinary medicine. High throughput is an important feature of protein chips. Antibodies against several diseases can be detected from only a single serum, and this factor is especially important for clinical research, which uses precious samples from rare and wild animals. Substrate selection and surface modification, as well as new substrate research and development, have become major research foci in the field of protein chips. Our present work indicates that iPDMS can provide a matrix for the detection of antibodies in chicken serum. In addition, the high sensitivity and specificity of protein microarrays render them powerful tools in disease detection [19, 20] and enable their use for determining antibody responses to infectious diseases [21]. In the future, we will print recombinant antigenic proteins of different avian viruses to achieve high-throughput detection results with the same serum.

## Conclusion

The nsp5 protein chips were developed for the detection of antibodies against IBV. These assays are comparable to the commercial IDEXX IBV Ab Test kit in terms of sensitivity and specificity. The RDT can generate results within 15 min and may be a suitable alternative to screen for the presence of IBV in chickens.

## Abbreviations

AIV: Avian influenza virus
CAV: Chicken anemia virus
CIT: Chemiluminescent immunoassay test
EDC: 1-Ethyl-3-(3-dimethylaminopropyl) carbodiimide
ELISA: Enzyme-linked immunosorbent assay
HRP-IgG: Horseradish peroxidase-labeled goat anti-chicken IgY
IB: Infectious bronchitis
IBDV: Infectious bursal disease virus
IBV: IB virus
iPDMS: initiator integrated poly(dimethylsiloxane)
MDV: Marek’s disease virus
NDV: Newcastle disease virus
NHS: N-hydroxysuccinimide
nsp5: Nonstructural protein 5
RDT: Rapid diagnostic test
SPF: Specific-pathogen-free
TMB: Tetramethylbenzidine
